# Prognostic impact of bone marrow iron and serum ferritin in patients with myelodysplastic syndromes

**DOI:** 10.1007/s00277-026-07106-w

**Published:** 2026-06-10

**Authors:** Annika Kasprzak, Felicitas Schulz, Kathrin Nachtkamp, Corinna Strupp, Maximilian Seidl, Martina Rudelius, Sascha Dietrich, Ulrich Germing, Norbert Gattermann

**Affiliations:** 1 Department of Hematology, Oncology and Clinical Immunology, University Hospital Düsseldorf, Düsseldorf, Germany; 2 Department of Pathology, University Hospital Düsseldorf, Düsseldorf, Germany; 3 Department of Pathology, Ludwigs-Maximilians-Universität München, Munich, Germany

**Keywords:** Ferritin, Iron overload, Prognosis, Bone marrow iron

## Abstract

We analysed 3,748 patients with myelodysplastic syndromes (MDS) to evaluate the prevalence and prognostic relevance of bone marrow (BM) iron accumulation at the time of initial diagnosis, its relationship with serum ferritin (SF), and its impact on overall survival (OS). Cytomorphological and/or histopathological BM iron assessment revealed that approximately 50% of patients already had increased iron stores at diagnosis. Elevated BM iron was significantly associated with more severe anaemia, higher SF levels, and lower transferrin levels. BM iron accumulation likely reflects prolonged ineffective erythropoiesis with increased intestinal iron absorption prior to transfusion dependency. Survival analyses demonstrated that increased BM iron adversely affected OS particularly in patients with MDS with increased blasts (MDS IB), whereas no significant effect was observed in lower-blast MDS. Multivariate Cox regression identified age and WHO subtype as the strongest prognostic factors overall, with SF as an independent predictor of OS; notably, in MDS IB, cytomorphological BM iron content emerged as the strongest independent prognostic parameter. These findings support a role for iron-induced oxidative stress in promoting genomic instability and disease progression in higher-risk MDS. We conclude that assessment of BM iron content and serum ferritin at diagnosis provides important prognostic information and should be incorporated into the initial evaluation of MDS patients.

## Introduction

Myelodysplastic syndromes (MDS) represent a heterogeneous group of clonal hematopoietic disorders characterized by ineffective haematopoiesis, dysplastic cell morphology, and an increased risk of progression to acute myeloid leukaemia (AML) [1]. Various factors contribute to the complexity of MDS, including the role of iron overload in disease progression and patient outcomes [2].

Iron, an essential element for many physiological processes, plays a pivotal role in cellular functions. When iron homeostasis is disrupted, as seen in conditions like MDS, this can lead to an excessive accumulation of iron in tissues, particularly the bone marrow [3]. Iron overload is a well-documented phenomenon in MDS patients, influenced by factors such as transfusion dependence, ineffective erythropoiesis, and altered iron metabolism [4].

The study presented below aims to provide an overview of the relationship between parameters related to iron metabolism in peripheral blood and bone marrow of patients with MDS, exploring various parameters that impact patient outcomes. An important and unique feature of the research cohort presented below is that the patients’ data were collected at the time of initial diagnosis, meaning all patients were transfusion naïve. This way we were able to rule out iatrogenic iron overload due to transfusion therapy. Understanding the intricate interplay between iron homeostasis and MDS is vital for developing therapeutic strategies to mitigate the detrimental effects of iron overload and improve the overall management of MDS patients [5]. Through an analysis of correlations between iron metabolism parameters in blood and bone marrow, this paper seeks to contribute to the growing body of knowledge regarding iron overload in the context of MDS [6].

## Methods

The patient cohort for our analyses comprised 3,748 patients from the Düsseldorf MDS Registry. We developed a data set with 884 variables containing disease- as well as patient-related data. Patient data and laboratory parameters at the time of initial diagnosis were obtained from patient records and the electronic hospital information system. In cases of unclear outcome, the respective local registration office was contacted, requesting information on the patients’ date of death. Bone marrow smears from the Düsseldorf MDS Registry Biobank were examined using an established and standardized iron staining method (Prussian Blue stain) to assess the extent of iron storage in bone marrow. The Prussian blue iron stain is a histochemical method used to detect iron in bone marrow specimens. It works by reacting ferric iron (Fe³⁺) with potassium ferrocyanide in an acidic environment, forming insoluble blue ferric ferrocyanide deposits. This allows visualization of iron stores primarily within macrophages and, to a lesser extent, within erythroid precursors. Bone marrow iron assessment is typically performed on aspirate smears and is reported using a semi-quantitative grading system. The most commonly used scale ranges from 0 to 6 (or modified 0 to 4 in some laboratories), reflecting the amount of stainable iron in macrophages. Grade 0 indicates complete absence of iron stores, while higher grades indicate progressively increased iron deposition, with grade 6 representing heavy iron overload. In addition to storage iron, the presence of sideroblasts (erythroblasts containing iron granules) may also be evaluated, particularly when assessing disorders of iron metabolism or ineffective erythropoiesis. This grading system helps clinicians distinguish between iron deficiency, normal iron stores, and iron overload conditions.

Iron staining results from bone marrow smears at the time of initial diagnosis were correlated with iron parameters in the peripheral blood. Bone marrow smears were available for all patients. For 1,250 patients bone marrow trephines were also available for histological analysis of iron content in the marrow.

The statistical analysis of the data was conducted using the statistical analysis software SPSS (Version 27.0). The aim was to determine correlations between laboratory parameters and the iron content in the bone marrow at the time of initial diagnosis as well as during the disease. To achieve this, contingency tables (chi-squared test) and cross-tabulations were employed. The Kaplan-Meier Method was used to analyse survival time and event free survival. Survival duration was calculated depending on laboratory parameters of iron homeostasis as well as iron content in bone marrow macrophages.

Finally, stepwise calculation of a regression model was performed using the Cox proportional Hazard Model. The stepwise regression allowed the identification of independent variables. A significance level of *p* < 0.005 was defined for all analyses.

## Results

### Patient characteristics

The research cohort included 3,748 patients (approximately 55% males). The median age at the time of initial diagnosis was 71 years (range 18–98 years). At the time of data cut-off, 2,599 patients (69.3%) had died, while 190 patients (5.1%) were lost to follow-up. The predominant MDS subtype, according to the WHO classification of 2022, was MDS with low blast count (MDS LB), diagnosed in 52.2% of patients. In addition, 25.1% of patients presented with MDS characterized by an increased blast count (MDS IB 1 (11.5%) or MDS IB 2 (13.6%)), while 7% were diagnosed as AML with myelodysplasia related changes (AML MRC), and 10.1% with chronic myelomonocytic leukaemia (CMML). MDS subtypes according to WHO 2022 are given in Table 1.

**Table 1:** Distribution of MDS subtypes according to WHO 2022 classification

WHO 2022 subtype	*N*	%
MDS LB	1,958	52.2
MDS LB and del(5q)	107	2.8
MDS IB 1	433	11.5
MDS IB 2	509	13.6
AML MRC	262	7.0
CMML 0	48	1.3
CMML 1	252	6.7
CMML 2	77	2.1
MDS U	25	0.7
MDS/MPN-RS-T	77	2.1
Total	3,748	100

Ferritin levels at time of initial diagnosis were available for 1,823 patients. Ferritin measurements at diagnosis were not available for all patients because ferritin testing was not routinely performed throughout the entire registry period. Comparison of patients with versus without available ferritin measurements did not reveal major differences in baseline clinical characteristics, suggesting that missing ferritin data were largely random. For 3,455 patients, hemoglobin levels (Hb) were on record. For 1,627 patients, all parameters required for determining the risk category according to the International Prognostic Scoring System Revised (IPSS-R) were accessible. Within this subgroup, an intermediate-risk type was found in 444 patients (27.3%). Notably, 23.1% of patients showed a very high-risk type, closely followed by 22.4% with a low-risk type, and 18.3% with high-risk disease. The smallest proportion comprised patients classified as very low risk (8.9%) (Table 2).

**Table 2:** Distribution of IPSS-R risk categories

IPSS-*R*	*N*	%
Very Low	145	8.9
Low	364	22.4
Intermediate	444	27.3
High	298	18.3
Very High	376	23.1
Total	1,627	100

### Cytomorphological and histological iron content in the bone marrow

Among all 3,748 patients, at least one stained bone marrow smear was available at the time of initial diagnosis. 51.7% of the patients (*n* = 1,938) exhibited a normal or even reduced iron content in the bone marrow macrophages. Conversely, an increased to excessively increased iron content was observed in 48.3% of the patients (Table 3).

**Table 3:** Cytomorphological iron staining

Cytological iron staining	*N*	%
Reduced	296	7.9
Normal	1,642	43.8
Increased	1,545	41.2
Excessively increased	265	7.1
Total	3,748	100

In 1,250 patients, bone marrow iron was assessed by histopathology in addition to cytomorphology, with 513 patients (41%) showing a reduced to normal iron content, and 737 patients (59%) showing an increased to excessively increased iron content (Table 4).

**Table 4:** Histological iron staining

Histological iron staining	*N*	%
No iron	95	7.6
Reduced	124	9.9
Normal	294	23.5
Increased	584	46.7
Excessively increased	153	12.3
Total	1,250	100

### Serum ferritin (SF) levels at the time of initial diagnosis

Serum ferritin was assessed in 2,672 patients at diagnosis. The mean value was 826 µg/l, and the median was 444 µg/l. We divided the patient cohort into three groups according to serum ferritin levels: normal (30–400 µg/l), decreased (< 30 µg/l), and increased (> 400 µg/l). The majority (*n* = 1,823) of the cohort demonstrated an elevated SF. Only 64 patients had decreased ferritin levels at initial diagnosis (Table 5).

**Table 5:** Categorization of serum ferritin distribution

Ferritin	*n*	%
Reduced	64	2.4%
Normal	785	29.4%
Increased	1,823	68.2%
Total	2,672	100%

### Correlation between bone marrow (BM) iron assessment and laboratory parameters at diagnosis

We correlated laboratory values at the time of initial diagnosis with BM iron content assessed by cytomorphology as well as histopathology. An interesting difference in average hemoglobin levels (9.3 g/dl vs. 8.8 g/dl, *p* < 0.001) was detectable between patients with normal or even decreased BM iron and those with increased BM iron according to cytomorphological analysis. More pronounced anemia was thus associated with increased BM iron. The result was similar when BM iron was assessed in core biopsies (Hb 9.5 g/dl vs. 8.9 g/dl, *P* < 0.001).

Individuals with increased BM iron on cytomorphology had significantly higher serum ferritin levels compared to those with decreased to normal BM iron. The mean SF among patients with cytomorphologically normal or decreased BM iron was 682.0 µg/l, suggesting that, actually, storage iron was already increased at the time of initial diagnosis. Conversely, patients with increased BM iron on cytomorphology had a mean ferritin level of 976.0 µg/l. The median SF also differed significantly between these groups (325.0 µg/l vs. 650.5 µg/l, *p* < 0.001). Similar results emerged from the analyses of core biopsies. Patients with decreased to normal BM iron had an average SF of 593.0 µg/l, whereas those with increased iron showed an SF of 909.0 µg/l. Once again, the median levels also differed significantly (287 versus 553 µg/l, *p* < 0.001).

Considering categorized SF levels, the correlation became even more apparent. On cytomorphology, 50.7% of patients with decreased to normal BM iron content had ferritin levels within the normal range, while 43.8% presented with increased SF. Conversely, among patients with increased BM iron, the majority (63.4%) showed increased SF, while 35.2% had physiological SF levels, and only 1.5% had decreased SF. This trend was consistent with histopathological assessment, where 54.3% of patients with decreased to normal BM iron had normal ferritin levels, while the majority (61.6%) of those with increased BM iron had elevated SF. Overall, the entire study cohort showed average SF levels higher than those in the general population (Table 6).

**Table 6:** Correlation between cytologically or histopathologically assessed BM iron and categorized serum ferritin

Serum ferritin categories	Cytomorphological iron decreased - normal		Cytomorphological iron increased		*p*-value
Reduced	51	5.6%	13	1.5%	< 0.001
Normal	465	50.7%	315	35.2%	
Increased	402	43.8%	568	63.4%	
Total	918	100%	896	100%	
	Histological iron decreased - normal		Histological iron increased		p-value
Reduced	23	7.6%	3	0.6%	< 0.001
Normal	165	54.3%	175	37.7%	
Increased	116	38.2%	286	61.6%	
Total	304	100%	464	100%	

Among patients with decreased to normal BM iron on cytomorphology, the mean transferrin level was 213 mg/dl (median 209 mg/dl), whereas patients with increased BM iron had a significantly lower mean transferrin level of 194.6 mg/dl (median 190 mg/dl) (*p* < 0.001). We did not find a significant correlation between the risk categories of the IPSS-R and BM iron content at initial diagnosis, neither on cytomorphological nor on histopathological analysis.

### Impact of serum ferritin and bone marrow iron on overall survival

We examined whether different SF levels had an impact on overall survival (OS) in patients in our study cohort. In patients with decreased SF at diagnosis, OS was 62 months, whereas patients with normal SF had a median survival of 36 months. Notably, patients with elevated SF showed an even shorter median OS of 28 months (*p* < 0.001).

**Fig. 1:**
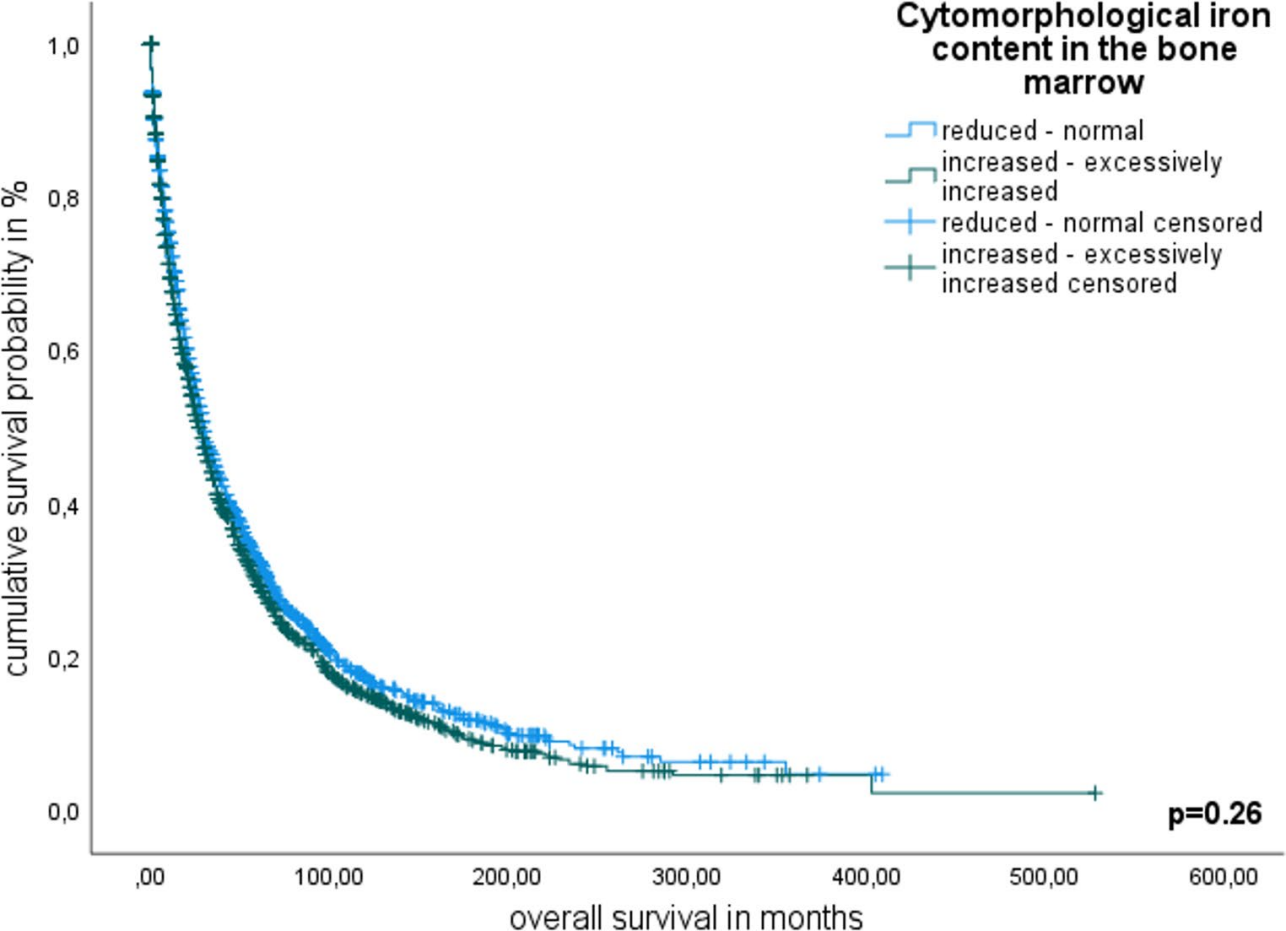
Overall survival according to cytomorphological iron content in the bone marrow

**Fig. 2:**
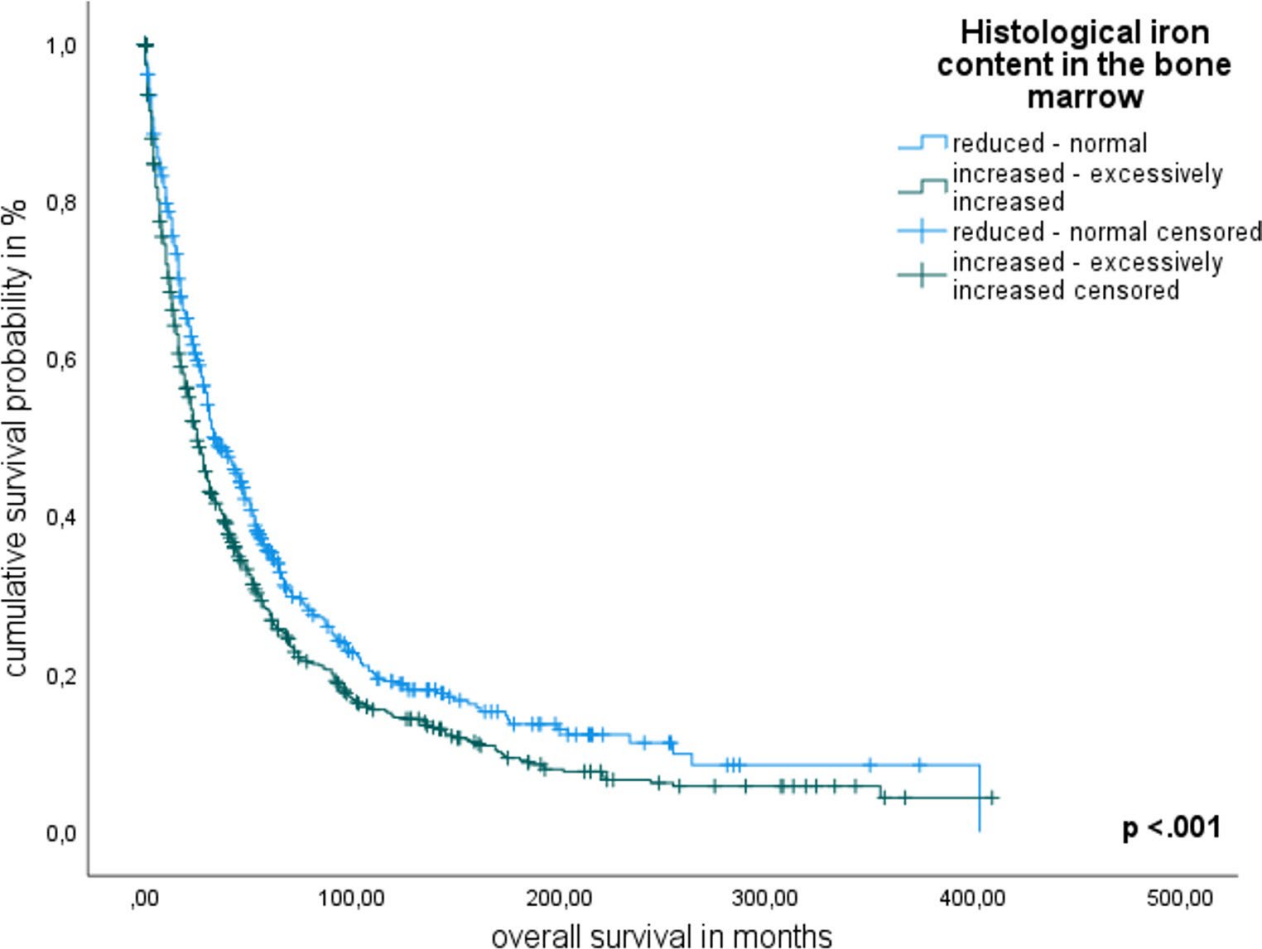
Overall survival according to histological iron content in the bone marrow

**Fig. 3:**
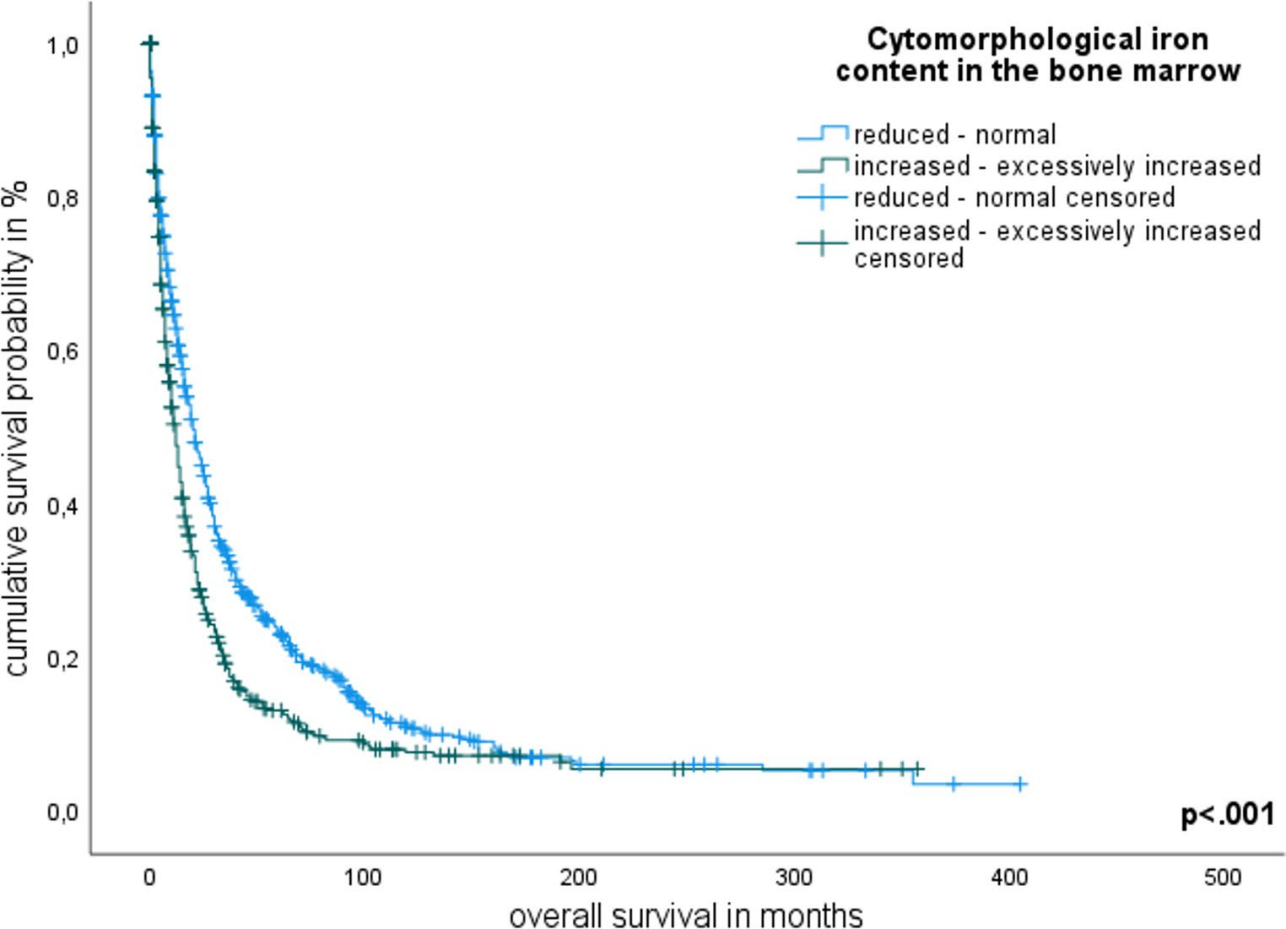
Overall survival according to cytomorphological iron content in the bone marrow in MDS with increased blast count

We also looked at the impact of BM iron content on overall survival. Patients with normal to decreased BM iron on cytomorphology had a median OS of 30 months. Median OS for patients with increased BM iron was not significantly different, namely 28 months (Fig. 1). BM iron on cytomorphology at the time of initial diagnosis did not have a significant impact on prognosis (*p* = 0.26). However, the median OS for patients with decreased to normal BM iron on histopathology was 34 months, while it was significantly shorter (25 months, *p* < 001) for those with increased BM iron (Fig. 2). To examine whether BM iron storage levels according to cytomorphological examination had an impact on survival in different risk groups, we divided our study population into patients with low risk and high-risk disease, according to blast count in the bone marrow. In patients with increased blast count (MDS IB), the median OS was 20 months for those with decreased to normal BM iron, and significantly shorter (12 months) for those with increased to excessively increased BM iron (*p* < 0.001, Fig. 3).

**Fig. 4:**
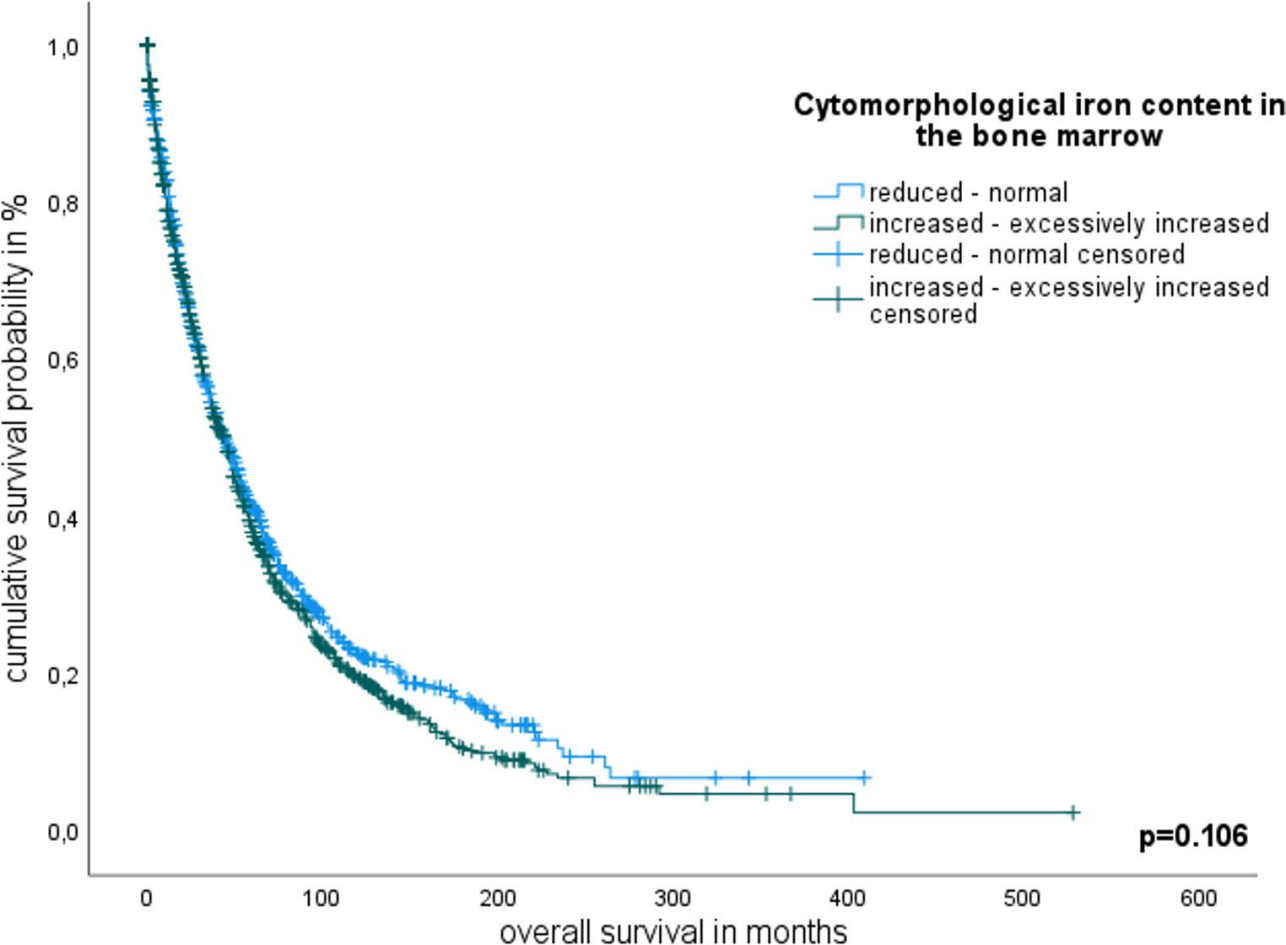
Overall survival according to cytomorphological iron content in the bone marrow in MDS with low blast count

Among patients with low blast count (MDS LB), the median OS was 45 months for those with decreased to normal BM iron, and similar (44 months) for those with increased to excessively increased BM iron (Fig. 4). Therefore, in patients with MDS LB, BM iron content assessed by cytomorphology at the time of diagnosis does not appear to have a significant impact on overall survival (*p* = 0.106).

**Fig. 5:**
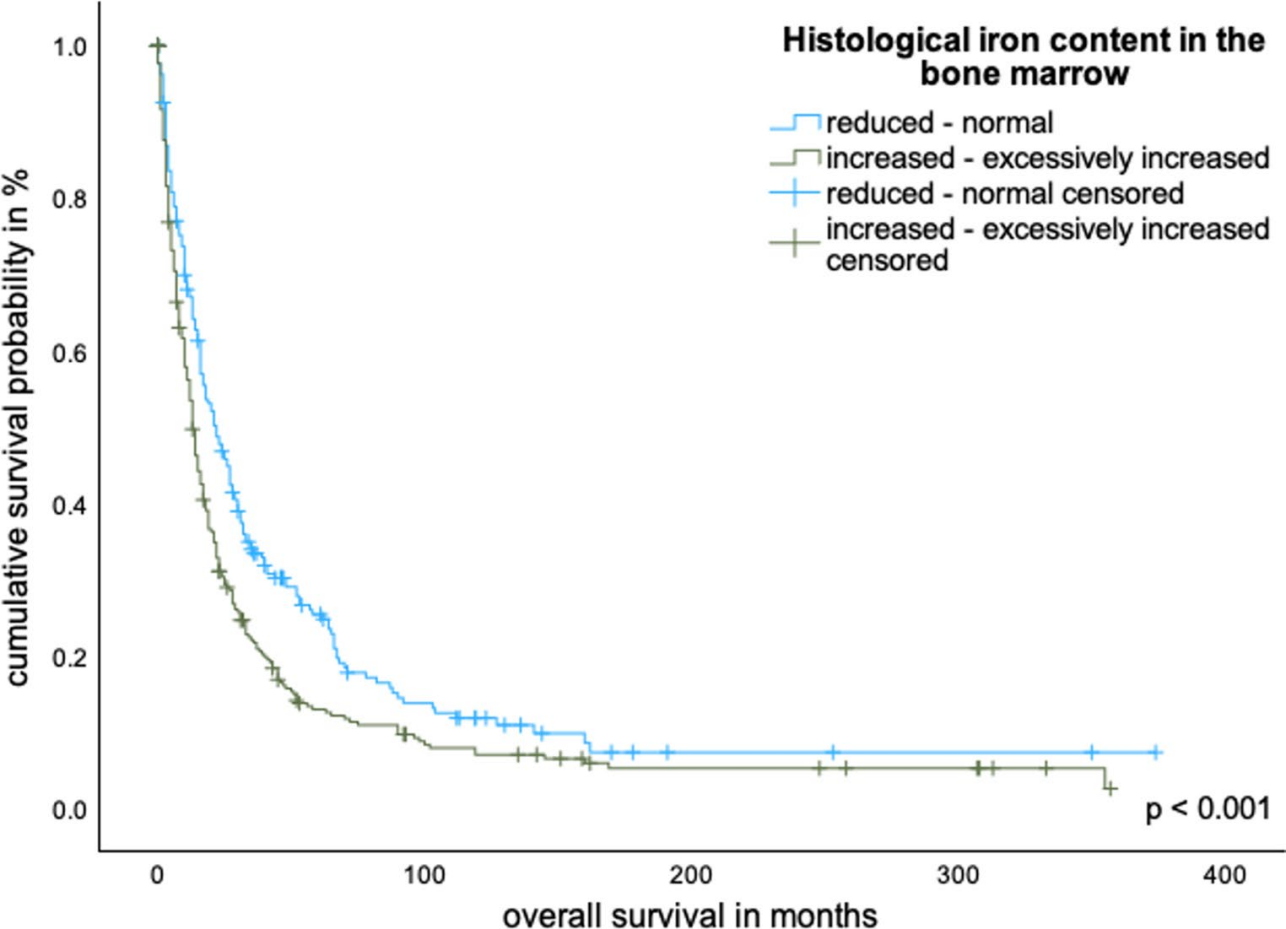
Overall survival according to histological iron content in the bone marrow in MDS with increased blast count

A similar analysis was performed for patients with MDS IB and LB regarding BM iron as assessed by histopathology. Patients with increased blast count and decreased to normal BM iron had a median OS of 22 months, while patients with increased to excessively increased BM iron had a significantly shorter median OS of 13 months (*p* < 0.001, Fig. 5).

**Fig. 6:**
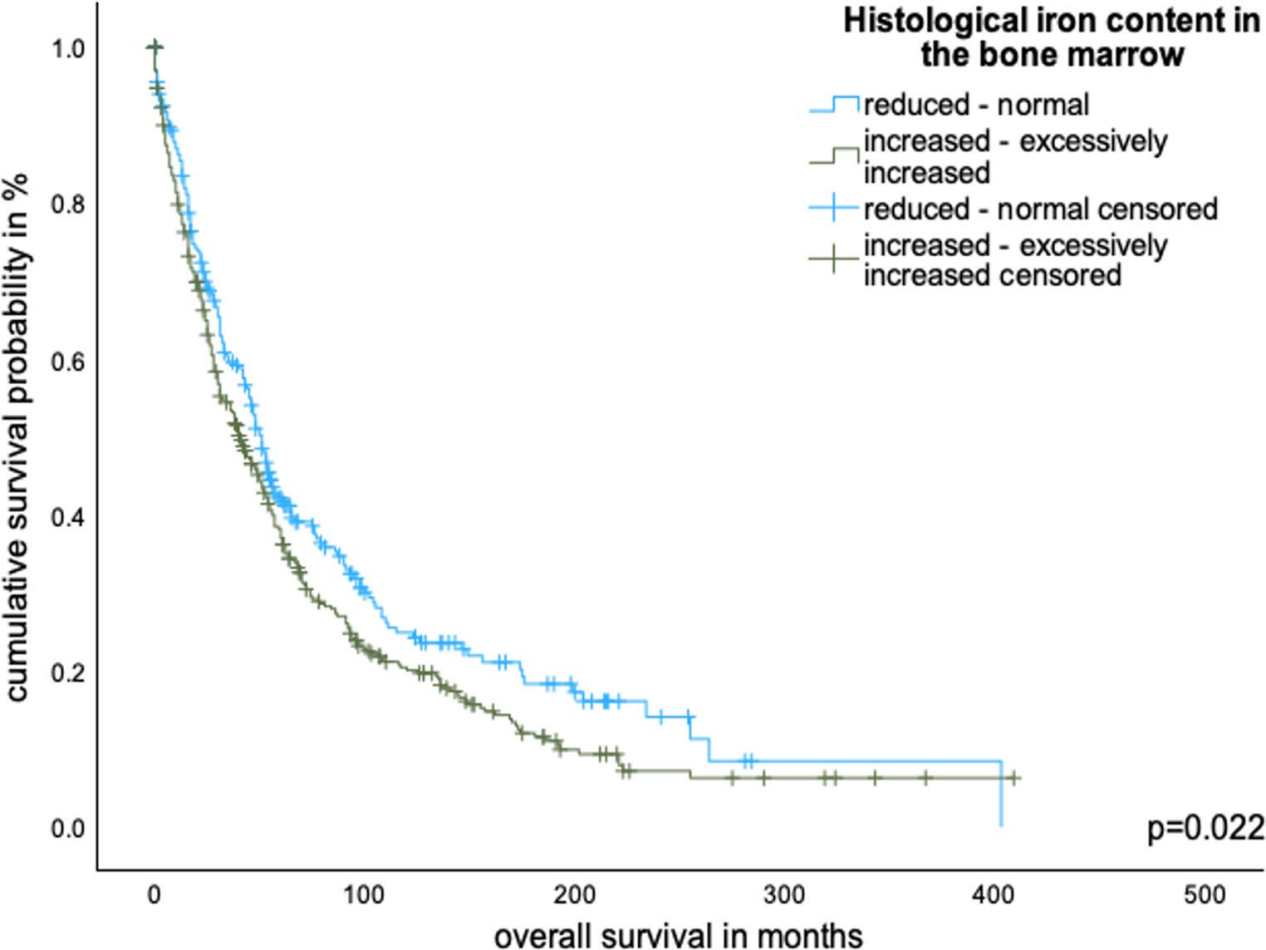
Overall survival according to cytomorphological iron content in the bone marrow in MDS with low blast count

In patients with low blast count, BM iron assessed by histopathology at initial diagnosis did not show a significant influence on prognosis (Fig. 6). Patients with decreased to normal BM iron had a median OS of 51 months, while patients with increased to excessively increased BM iron had a median OS of 41 months (*p* = 0.022).

Additional subgroup analysis of patients with MDS and ring sideroblasts (RS), restricted to low- and intermediate-risk IPSS-R categories and excluding cases with increased blast count, demonstrated that elevated serum ferritin levels were associated with inferior overall survival. Patients with increased ferritin showed a median overall survival of 58 months compared with 74 months in patients with normal ferritin levels (*p* = 0.031).

### Impact of serum ferritin and BM iron on prognosis, adjusted for age and WHO subtype

We investigated the impact of SF levels, as well as cytomorphologically and histopathologically assessed BM iron on overall survival, adjusted for age and WHO subtype (categorized into MDS IB and LB), using Cox regression analysis. Cox regression analysis for the entire study cohort showed that stratification according to low versus elevated blast count, as well as between different SF levels at diagnosis, yielded prognostically relevant parameters that were independent of each other (Table 7). Elevated ferritin levels at diagnosis had an unfavourable impact on the prognosis of MDS patients, regardless of their WHO subtype. BM iron content assessed by cytomorphology or histopathology at diagnosis were not confirmed as independent prognostic factors.

**Table 7:** Multivariate cox regression analysis for the whole study cohort including WHO Subtype, ferritin levels, age and histological as well as cytomorphological iron content in the bone marrow

Multivariate analysis of the total study cohort	*p*-value	Exp(B)	
MDS IB vs. LB		< 0.001	2.593
Ferritin levels at initial diagnosis	< 0.001		
Decreased – normal ferritin levels	0.306	1.271	
Increased – excessively increased ferritin levels	0.025	1.690	

In contrast to the entire study group, the subgroup of MDS patients with low blast count did not show a significant impact of SF on prognosis. Again, BM iron content assessed by cytomorphology or histopathology at diagnosis were not confirmed as independent prognostic factors (Table 8).

**Table 8:** Multivariate cox regression analysis for patients with MDS LB including ferritin levels, age and histological as well as cytomorphological iron content in the bone marrow

Multivariate analysis of MDS LB patients	*p*-value	Exp(B)
Ferritin levels as initial diagnosis	0.070	
Decreased – normal ferritin levels	0.773	1.098
Increased – excessively increased ferritin levels	0.260	1.438

Focusing on MDS IB, the strongest independent parameter was, in contrast to our previous analyses, an increased cytomorphological iron content in the bone marrow. This parameter was superior to SF at initial diagnosis, which had a much weaker prognostic impact (Table 9).

**Table 9:** Multivariate cox regression for patients with MDS IB including ferritin levels, age and histological as well as cytomorphological iron content in the bone marrow

Multivariate analysis of MDS LB patients	*p*-value	Exp(B)
Ferritin levels as initial diagnosis	0.048	
Decreased – normal ferritin levels	0.702	1.145
Increased – excessively increased ferritin levels	0.210	1.577
Increased cytomorphological iron content	< 0.001	1.593

## Discussion

In this study, we analysed 3,748 MDS patients to see how cytomorphological and histopathological assessment of iron content in bone marrow macrophages correlate with serum ferritin levels, and how both parameters, measured at the time of initial diagnosis, correlate with overall survival. Bone marrow trephine biopsies for histopathological examination at the time of diagnosis were available for 1,250 patients. We focused on data generated at the time of initial diagnosis to minimize the influence of blood transfusions and other therapies that might influence iron homeostasis.

We found that on cytomorphological as well as histopathological analysis approximately half of the patients showed increased bone marrow iron at the time of diagnosis, before the first transfusion was administered. Those with increased or massively increased BM iron had more pronounced anemia than patients with reduced to normal BM iron. Patients with increased BM iron presented with significantly higher serum ferritin levels than patients with reduced to normal BM iron. Patients with increased or massively increased BM iron also showed lower transferrin levels. The latter were inversely correlated with iron content in bone marrow macrophages.

Patients with MDS typically experience refractory anemia. Due to ineffective haematopoiesis in the bone marrow, this type of anemia does not respond to iron supplementation. However, ineffective erythropoiesis causes downregulation of hepcidin production in the liver, resulting in increased iron absorption in the intestine. In the long term, enhanced duodenal iron uptake leads to excess total body iron, which may already be present at the time of MDS diagnosis. Elevated BM iron at diagnosis is therefore attributable to a prolonged phase of increased duodenal iron absorption due to ineffective erythropoiesis, probably associated with a certain degree of anemia. With disease progression, the ensuing transfusion dependency markedly contributes to iron accumulation.

In this context, it is conceivable that a subset of patients with elevated serum ferritin at the time of diagnosis had already experienced a prolonged phase of moderate, clinically underestimated anemia prior to formal MDS diagnosis. These patients may not initially have been classified as low-risk MDS, and a high-risk diagnosis may only have been established at a later stage. In such cases, iron accumulation could represent a subclinical precursor reflecting ineffective erythropoiesis and increased intestinal iron absorption long before overt disease progression or transfusion dependency became apparent. Thus, elevated ferritin at diagnosis may partly capture a hidden disease trajectory preceding formal risk stratification.

In 2020, Pilo et al. conducted a study examining iron accumulation in 114 patients with MDS. The iron content in the bone marrow was characterized as follows: grade 0 denoted absent iron storage with increasing iron content up to grade 3, which indicated increased iron content. None of the patients had received prior iron chelation therapy. Twenty-seven patients were categorized as grade 1, 31 patients as grade 2, and 56 as grade 3. As in our study, nearly 50% of the patients presented with increased iron [7]. Median Hb at the time of MDS diagnosis was 10.7 g/dl in patients with grade 1 iron content, 9.8 g/dl in the group with grade 2, and 8.6 g/dl in the iron storage group 3. Thus, Pilo et al. also demonstrated a correlation between increased BM iron and anemia.

Song et al. (2016) retrospectively analysed iron parameters in 94 newly diagnosed MDS patients between June 2015 and March 2016. They also found increased iron storage in 52 patients (55.3%) at the time of initial diagnosis [8].

We assessed the impact of BM iron content at the time of diagnosis on overall survival, using the Kaplan-Meier estimator. We found that BM iron assessed by cytomorphology did not significantly affect OS. While this was true for patients with MDS LB, patients with IB had a significantly shorter OS when they showed increased BM iron.

A similar result was obtained with bone marrow histopathology. In the entire study cohort, BM iron significantly influenced OS. While this influence was not clearly demonstrable in MDS LB, patients with MDS IB showed a significant unfavourable impact of elevated BM iron content on overall survival.

As clones in MDS with elevated blast count are typically less stable and more prone to clonal evolution, patients with MDS IB have a worse prognosis per se. However, since we observed that increasing BM iron accumulation affects OS especially in MDS IB, we suggest that iron-related oxidative stress exacerbates clonal genetic instability and thus contributes to shortened overall survival.

Experimental studies have shown that iron overload contributes to stromal dysfunction in the bone marrow, increases genomic instability, and accelerates clonal evolution, thus leading to worse OS. Westhoven et al. found that SF levels above the normal range contribute to genetic instability in MDS. Hematopoietic stem cells that are already affected by genomic instability appear to be particularly vulnerable to oxidative stress induced by iron overload [9].

In our study, the independent prognostic impact of BM iron and SF on the overall survival of MDS patients was investigated through forward Cox regressional analysis. The analysis included WHO subtype, age, SF levels, as well as cytomorphological and histopathological iron content in bone marrow macrophages at the time of initial diagnosis. As anticipated, WHO subtype and the patient’s age at initial diagnosis emerged as the most significant independent parameters in the overall cohort. Serum ferritin proved to be the second strongest independent parameter. MDS patients with elevated SF at initial diagnosis thus have a worse prognosis compared to those with reduced or normal SF.

Surprisingly, patients with MDS IB diverged from both the overall cohort and patients with MDS LB by showing increased cytomorphological BM iron content as the strongest independent parameter.

When Gattermann et al. analysed iron overload in patients with MDS [10], they demonstrated a dose-dependent negative impact on OS. While higher SF levels may largely reflect higher transfusion requirements due to more severe bone marrow disease (thereby explaining shortened survival), Malcovati et al. demonstrated in a multivariate analysis that SF is an independent prognostic marker. For each increase in SF by 500 µg/l above a threshold of 1,000 µg/l, the risk of death increased by 30%. Transfusion dependency and SF were independent prognostic factors for overall survival [11].

Ineffective haematopoiesis is partly attributable to an altered bone marrow microenvironment. The bone marrow stroma is important for quality, function, and renewal of hematopoietic stem cells. Mesenchymal stromal cells (MSCs) support physiological haematopoiesis. Huang et al. investigated the effects of iron overload on mesenchymal stromal cells in 40 patients with high-risk MDS and 13 patients with MDS/AML. A serum ferritin level above 1,000 ng/ml was considered as indicating iron-overload. The study showed that iron overload reduced the number of mesenchymal stromal cells and negatively affected their proliferation and differentiation. Moreover, iron overload inhibited the gene expression of signalling molecules in mesenchymal stromal cells that are involved in blood formation. Iron overload caused increased levels of reactive oxygen species (ROS), thereby triggering apoptosis in mesenchymal stromal cells, which in turn may contribute to the aggravation and progression of myelodysplastic syndromes. The effects of iron overload on mesenchymal stromal cells were be mitigated by the administration of antioxidants and iron chelators [12].

To conclude, the present study demonstrates that BM iron content at diagnosis has prognostic influence on the course of disease, especially in higher risk MDS with elevated blast count. Approximately 50% of MDS patients already have increased BM iron at the time of initial diagnosis. A significant positive correlation was demonstrated between BM iron and serum ferritin, and a significant negative correlation between BM iron and hemoglobin. We also show that SF levels can be used as an independent prognostic factor in MDS patients.

Based on this study, prognostic evaluation at initial diagnosis should include an assessment of BM iron through cyto- and histo morphological analysis (iron staining) and measurement of serum ferritin.

## Data Availability

The datasets generated and/or analyzed during the current study are available from the corresponding author upon reasonable request due to institutional restrictions.
